# Palliative and end-of-life care research in Scotland 2006–2015: a systematic scoping review

**DOI:** 10.1186/s12904-017-0266-0

**Published:** 2018-01-26

**Authors:** Anne M. Finucane, Emma Carduff, Jean Lugton, Stephen Fenning, Bridget Johnston, Marie Fallon, David Clark, Juliet A. Spiller, Scott A. Murray

**Affiliations:** 1 Marie Curie Hospice Edinburgh, 45 Frogston Road West, Edinburgh, EH10 7DR UK; 2 Marie Curie Hospice Glasgow, 133 Balornock Road, Glasgow, G21 3US UK; 3 Western General Hospital, Crewe Road South, Edinburgh, EH4 2XU UK; 4 Florence Nightingale Foundation, Clinical Nursing Practice Research, School of Medicine, Dentistry & Nursing, College of Medical, Veterinary & Life Sciences, University of Glasgow and NHS Greater Glasgow and Clyde, 57-61 Oakfield Avenue, Glasgow, G12 8LL UK; 5 Institute of Genetics and Palliative Medicine, University of Edinburgh, Western General Hospital, Edinburgh, EH4 2XR UK; 6 School of Interdisciplinary Studies, University of Glasgow, Bankend Road, Dumfries, DG1 4ZL UK; 7 Centre for Population Health Sciences, The Usher Institute of Population Health Sciences and Informatics, University of Edinburgh, Old Medical School, Teviot Place, Edinburgh, EH8 9AG UK; 8 School of Medicine, Nursing and Healthcare, University of Glasgow, 59 Oakfield Avenue, Glasgow, G12 8LL UK

**Keywords:** Palliative, Research, Scoping review, End-of-life, Hospice, Cancer, Non-cancer, Policy, Knowledge exchange

## Abstract

**Background:**

The Scottish Government set out its 5-year vision to improve palliative care in its Strategic Framework for Action 2016–2021. This includes a commitment to strengthening research and evidence based knowledge exchange across Scotland. A comprehensive scoping review of Scottish palliative care research was considered an important first step. The aim of the review was to quantify and map palliative care research in Scotland over the ten-year period preceding the new strategy (2006–15).

**Methods:**

A systematic scoping review was undertaken. Palliative care research involving at least one co-author from a Scottish institution was eligible for inclusion. Five databases were searched with relevant MeSH terms and keywords; additional papers authored by members of the Scottish Palliative and End of Life Care Research Forum were added.

**Results:**

In total, 1919 papers were screened, 496 underwent full text review and 308 were retained in the final set. 73% were descriptive studies and 10% were interventions or feasibility studies. The top three areas of research focus were services and settings; experiences and/or needs; and physical symptoms. 58 papers were concerned with palliative care for people with conditions other than cancer – nearly one fifth of all papers published. Few studies focused on ehealth, health economics, out-of-hours and public health. Nearly half of all papers described unfunded research or did not acknowledge a funder (46%).

**Conclusions:**

There was a steady increase in Scottish palliative care research during the decade under review. Research output was strong compared with that reported in an earlier Scottish review (1990–2005) and a similar review of Irish palliative care research (2002–2012). A large amount of descriptive evidence exists on living and dying with chronic progressive illness in Scotland; intervention studies now need to be prioritised. Areas highlighted for future research include palliative interventions for people with non-malignant illness and multi-morbidity; physical and psychological symptom assessment and management; interventions to support carers; and bereavement support. Knowledge exchange activities are required to disseminate research findings to research users and a follow-up review to examine future research progress is recommended.

**Electronic supplementary material:**

The online version of this article (10.1186/s12904-017-0266-0) contains supplementary material, which is available to authorized users.

## Background

Palliative care research has an essential role in informing evidence based clinical practice, service development, education and policy. Acknowledging this, the Scottish Government included a commitment to supporting research in its national strategy for palliative and end of life care for 2016–2021 [1]. This strategy sets out the vision that, by 2021, everyone in Scotland who needs palliative care will have access to it, and it identifies ten commitments to support a range of improvements in the delivery of palliative care. The fifth commitment focuses on research and advocates the establishment of a Scottish Research Forum in Palliative and End of Life Care to strengthen research and co-ordinate knowledge exchange across the country. A report published by the Health and Sport Committee recommended that a systematic review be conducted to map the evidence base and explore the vitality of Scottish palliative care research [2]. This recommendation was included in the evidence summary report underpinning the national palliative care strategy [3].

Country-wide reviews of palliative care research are typically motivated by recognition of the importance of research evidence in supporting decision-makers to meet the challenges of palliative care in the twenty-first century. Such reviews reveal the quantity and foci of research in a geographical context and provide a baseline against which future research output can be measured. They highlight strengths that can be harnessed and research gaps that may need to be addressed, either regionally or nationally. This process results in a database of research publications and findings which can be shared with non-academic research users to inform service development, innovation, policy and practice in the geographical context under study. This in turn can provide a basis for knowledge exchange activities between the academic community and research users based in clinical, educational and policy settings.

Country-wide reviews of palliative care research have been conducted previously in Ireland, Sweden and Scotland. Following the launch of the All Ireland Institute of Hospice and Palliative Care (AIIHPC), Ireland conducted a review of all palliative care research spanning 2002 to 2012 with the aim of describing the research context and setting priorities for the future [4]. Similarly, in Sweden, a review covering the period 2007–2012 examined the type of research conducted, growth over time and changes in foci compared to a previous baseline review [5]. In Scotland, an unpublished review examined all palliative care research conducted between 1990 and 2005, to identify gaps and set priorities [6]. That review identified 44 research publications over a 15-year search period, with key themes relating to models of palliative care provision, symptom control, spiritual issues and education.

The present scoping review aimed to identify all Scottish palliative care research published over the ten-year period from January 2006 to December 2015 and to map this into broad thematic areas. Research questions were:

1. How many Scottish research papers were published over the 10-year period from 2006 to 2015? How did annual numbers vary over the ten year period?
2. What were the areas of focus for palliative care research in Scotland?
3. What types of studies have been undertaken, what designs have been used, and what are the characteristics of the study populations?
4. What strengths and gaps exist?

## Methods

### Design

A systematic scoping review was conducted to address the research questions identified. Systematic scoping reviews are particularly useful for mapping broad areas of research and allow inclusion of diverse types of evidence to inform practice in the field [7]. Systematic scoping reviews are conducted using systematic searches and follow an a priori search protocol which may be adjusted iteratively. Quality appraisal is not conducted in systematic scoping reviews given that the focus is on comprehensively mapping the area of interest and often results in the identification of many research studies using diverse methods. Follow-up work may involve quality appraisal and evidence synthesis of specific areas identified.

### Search strategy

Five databases were searched between February and March 2016: Medline, Embase, Cinahl, PsychInfo and the Cochrane Pain Palliative and Supportive Care Database. The search consisted of a combination of search terms relevant to palliative care in conjunction with search terms relevant to a Scottish context. Terms to retrieve research relevant to palliative care included ‘palliative’, ‘terminal’, ‘hospice’, ‘dying’, ‘end of life’, ‘last year of life’, ‘bereavement’, ‘place of death’, ‘supportive care’ as well as combinations of terms such as ‘end-stage’ in conjunction with ‘heart’ or ‘liver’ or ‘kidney’. To identify research conducted by Scotland-based researchers, search terms included ‘Scotland’ or ‘Scottish’ in the title or abstract, or contained the name or part-name of a Scottish research institution in the ‘Institution’ field (e.g. Edinburgh, Glasgow, Stirling, Dundee, Strathclyde, Aberdeen, Highlands). Search terms included MeSH terms and keywords as appropriate depending on the database searched. See Appendix 1 for the Medline search strategy.

To supplement the systematic search, publication lists of well-established palliative care researchers based in Scotland were examined, and researchers involved in the Scottish Palliative and End of Life Care Research Forum were invited to highlight any potentially eligible publications for the review.

### Inclusion and exclusion criteria

The present review includes any publication relating to palliative care in accordance with the WHO definition. The WHO describes palliative care as *“..an approach that improves the quality of life of patients (adults and children) and their families who are facing problems associated with life-threatening illness. It prevents and relieves suffering through the early identification, correct assessment and treatment of pain and other problems, whether physical, psychosocial or spiritual*” [8]. Research studies focused on supportive and symptom focused care for people with life-threatening conditions were included, whereas studies on curative, restorative or disease specific aspects of care were excluded.

For inclusion papers needed to meet the following criteria: (i) at least one author based at a Scottish institution, (ii) describe the results of research or service evaluation/audit underpinned by research methods, (iii) English language and (iv) peer reviewed. Papers reporting research on disease-modifying or active treatment were excluded, as were protocols, guidelines, case reports, commentaries, editorials, conference abstracts and pure service evaluation/audit. Research papers conducted by Scotland based authors where data was collected overseas were included. Papers reporting data collected in Scotland, where there were no Scotland based co-authors were excluded, as these would be impossible to identify systematically. Reasons for excluding papers at the full text review stage were documented.

### Data extraction

All citations were exported to Endnote and duplicates were removed. A data extraction protocol was developed and used by four data extractors (AF, JL, EC and SF) to extract key information and categorise research into key themes. The data extracted from each included paper was as follows: author(s), year of publication, journal, title, international co-author(s), region(s) where data was collected (NHS Board area or country if outside Scotland), aim, study population, setting, broad study description, study type, type of evidence, methods of data collection, findings, funder, research themes (up to 3 themes were identified for each paper), audience, focus on cancer or noncancer.

To categorise research, a set of possible themes were initially identified from previous review studies [4, 6]. These included symptom management, services and settings, bereavement, communication and education. During the data extraction process, additional themes were identified and added to the list of possible themes. For instance, symptom management was further divided into a physical and psychological/psychosocial theme given the large number of papers, and additional themes such as ‘co-ordination’, ‘carers’ and ‘quality of life’, emerged and were added to the list of potential themes. For each paper, up to three themes were identified – every paper had at least one theme, and some had two or three. Data was extracted on an Excel spreadsheet. After initial extraction by one member of the research team, the extracted data was cross-checked by another team member (AF, JL, EC or SF), so all data extracted was double checked. Excluded papers and reasons for exclusion were also double checked. Where there was uncertainty or disagreement between extractors, the wider team were invited to contribute an opinion enabling a consensus to be reached.

## Results

**Fig. 1:**
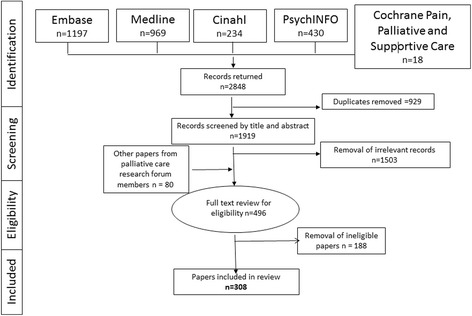
PRISMA flow chart highlighting the search strategy

Electronic searches yielded 2848 citations which were imported into Endnote. Duplicates were removed resulting in 1919 citations. These were screened by title and abstract by AF and JL, and 1503 irrelevant records were removed. Potentially relevant papers from the Scottish Research Forum for Palliative and End of Life Care that had not already been identified were added (*n* = 80). In total, 496 publications were identified for full text review. Of these, 188 papers were subsequently excluded. The reasons for exclusion were: i) not focused on palliative care (*n* = 69); ii) editorial/comment/discussion/opinionpaper (*n* = 54); iii) no Scotland based co-author (*n* = 15); iv) focus on disease modifying or active treatment (*n* = 14); iv) other (e.g. conference abstract, not research, clinical case report, book chapter) (*n* = 36). The search process is outlined Fig. 1.

### Number of Scottish palliative care research publications (2006–15)

**Fig. 2:**
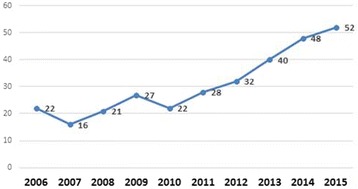
Number of palliative care research papers published by Scotland based researchers by year (*n* = 308)

**Fig. 3:**
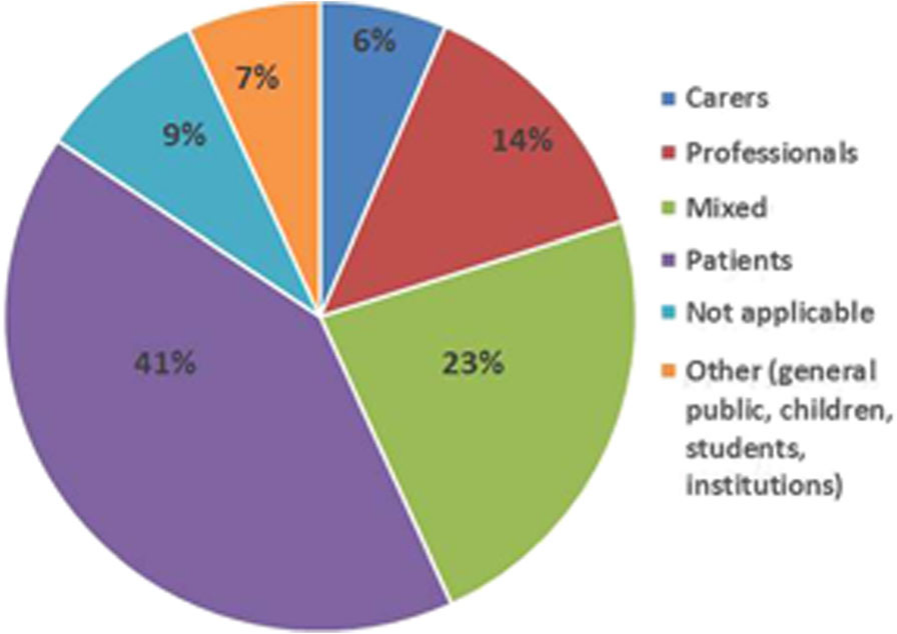
Participants in Scottish palliative care research studies published 2006–15 (n = 308)

**Fig. 4:**
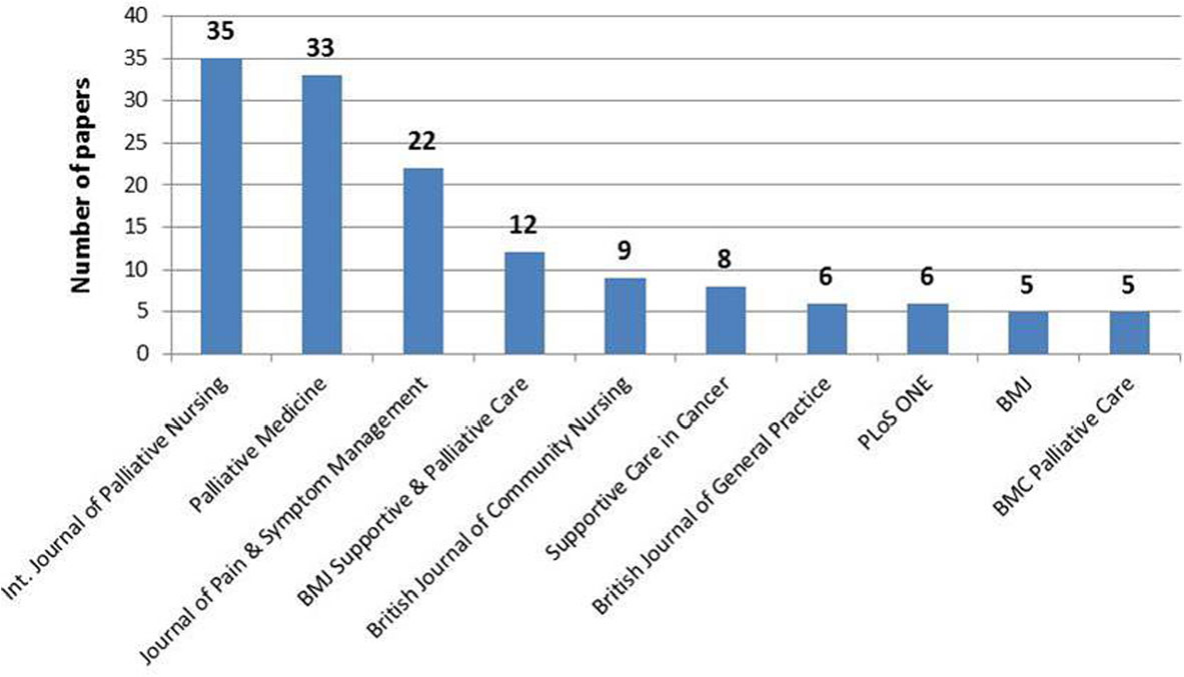
Top 10 most popular journals for palliative care research in Scotland (2006–15)

The search yielded 308 Scottish palliative care research publications. Findings from all 308 papers are summarised in Additional File 1. There was an upward trend in the number of papers published over the ten-year period 2006 to 2015 (Fig. 2). Annual output increased from 22 palliative care research papers published in 2006 to 52 papers in 2015. About half (51%) of all papers involved *only* authors based in Scotland, 28% involved authors from Scotland and the wider UK, while one-fifth (21%) involved at least one author based outside the UK. In terms of study participants, most papers involved patients only (*n* = 127), mixed participant groups (*n* = 71) or healthcare professionals only (*n* = 42). Only four papers involving research with children were identified (Fig. 3). Papers were published in 125 national and international journals, the most popular of which were the International Journal of Palliative Nursing (*n* = 35), Palliative Medicine (*n* = 33) and the Journal of Pain and Symptom Management (*n* = 22) (Fig. 4).

### Research settings

We examined the settings in which data was collected. Nearly one-quarter of studies involved data collection in a hospital setting (23%) and just over one-fifth involved data collection across a mix of settings (21%). Thirty papers (10%) reported data collected in hospices and 15 papers involved data collection in the participants home, though home was also a location for data collection in mixed-setting studies. Only 3% (8 papers) reported data collection specifically in care homes. Other settings included GP practices (13 papers) and other community settings. A significant number of papers did not specify the data collection setting (43 papers). For review and secondary analyses papers, a specific setting was not identified or not applicable.

### Types of studies undertaken

**Fig. 5:**
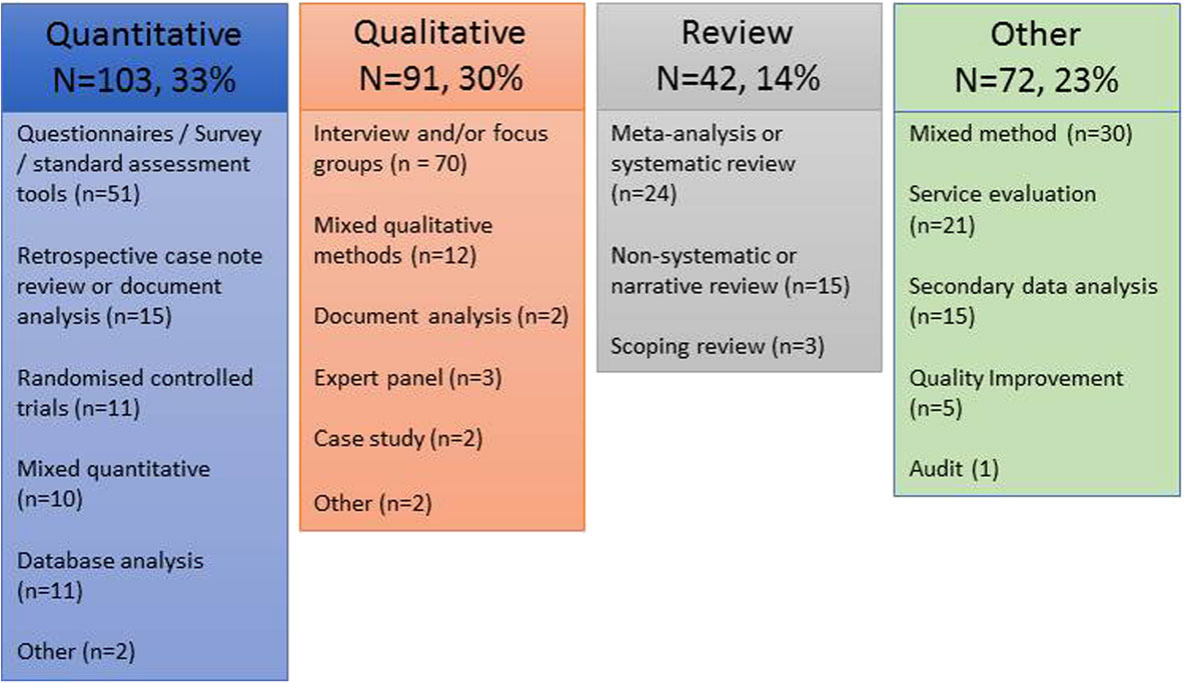
Methods reported in Scottish palliative care research publications (n = 308)

In terms of broad study description, one-third of papers were quantitative, 30% were qualitative and 14% were reviews. The remaining 23% included mixed method designs, secondary data analysis, service evaluation and quality improvement (Fig. 5). Twelve papers reported studies using RCT designs - eleven of these were quantitative studies and one was a mixed method study. Further examination of study type revealed that 73% were primarily descriptive studies (*n* = 225), and 10% reported findings from intervention or feasibility/pilot studies (*n* = 31). Other study types included service evaluation, methodological studies, health economics and implementation studies or guideline development (*n* = 52).

### Study populations

**Fig. 6:**
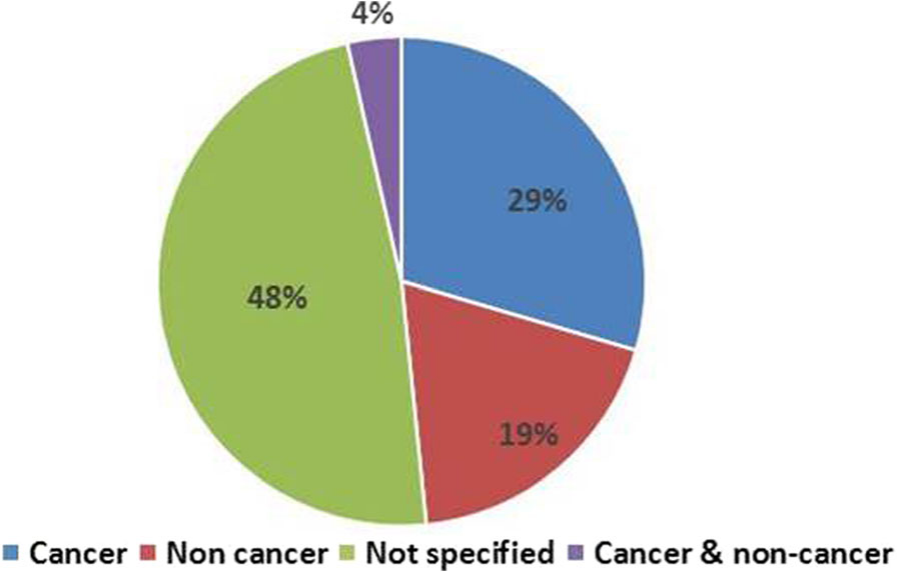
Diagnosis of participants in published studies (n = 308)

We examined whether the research related to cancer or non-cancer (Fig. 6). Nearly half of the papers did provide details on the study population. Ninety-one papers were focused on cancer and 58 papers (19%) were focused on palliative care for people with other diagnoses (e.g. heart failure, stroke, COPD, advanced liver disease, frailty/dementia). A small number of comparison papers (4%) included data on cancer and non-cancer groups. The number of research papers on palliative care for people with non-malignant disease increased over the decade, and in 2015, a similar number of cancer focused and non-cancer focused papers were published (11 non-cancer and 10 cancer focused).

### Areas of focus

**Fig. 7:**
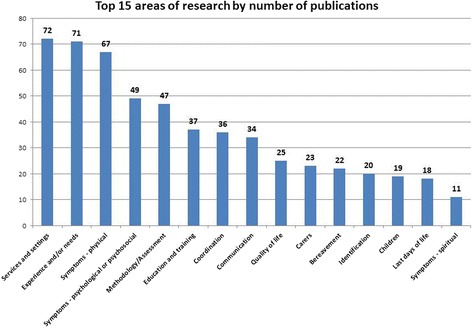
Top 15 themes relating to Scotland based palliative care research

The themes most frequently described in the 308 papers were services and settings (72 papers), experiences and/or needs (71 papers), and physical symptoms (67 papers). Other areas of focus included psychological or psychosocial issues, methodology and assessment, education and training, co-ordination, communication, quality of life, carers, bereavement and identification of people for a palliative approach. There were a modest number of papers on children, last days of life and spiritual symptoms; but very few on out-of-hours care (*n* = 9), health economics (*n* = 10), public health approaches (*n* = 7) and eHealth (*n* = 8) (Fig. 7). There were no papers specifically focused on social care.

A large number of studies on services and settings have provided evidence on the need for, and delivery of, palliative care in hospital and community settings. Scottish research has shown that, on any given day, a significant proportion of hospital inpatients are in the last year of life [9], and there are many studies exploring palliative care in hospital settings [10–14]. This work complements a growing body of research in primary care examining the role of non-specialists delivering palliative care in the community [15–18]. Research on experiences and needs has also been a core focus, and there is now substantial evidence around diverse illness trajectories following diagnosis of an advanced progressive illness, each associated with multidimensional needs of patients and carers [19–22]. Several studies have adopted longitudinal designs to explore changing experiences and needs from diagnosis to bereavement [23–25]. Evidence from Scottish studies has highlighted key points at which patient and carer distress is greatest and when support is most needed [16, 22]. Many studies have taken a multi-perspective approach gathering data from patients, carers and healthcare professionals, enabling data triangulation and a holistic perspective on care. Physical symptoms have been a key area of research activity in Scotland. Research on physical symptoms has focused, in particular, on pain [26–29], cachexia [30–38] and delirium [39–44]. Pain and cachexia has been underpinned by a translational research programme but the outputs from this are not addressed in this paper.

### Sources of funding

We searched the acknowledgements of each paper for any reference to funders. There was no reference to funders in 142 papers (46%). Of these, 99 papers did not refer to any source of funding for the research, while a further 43 papers included a statement that the research was unfunded. Research funded solely by charities or a mix of charities was described in 14% of papers. Mixed funding sources were acknowledged in 18% of papers. These included partnerships between charities and the NHS, charities and a Research Council, charities and the government as well as funding where a co-authors substantive employment was acknowledged as part of the funding. Other funders included the NHS, NIHR, Chief Scientist Office (CSO), European Union and Research Councils.

## Discussion

The findings from this scoping review clearly show a considerable increase in palliative care research in Scotland over the 10-year period from 2006 to 2015. Overall 308 papers were identified - seven times as many publications as identified in a previous Scottish palliative care research review covering a 15-year period from 1990 to 2005 [6]. Output was also strong compared with that reported in a review of palliative care research in Ireland over a similar 10-year period [4]. The Irish review identified about half the number identified in the present Scottish review (151 research publications between 2002 and 2012). Taken together our review suggests that Scotland performs very well in terms of volume of palliative care research publications.

In Scotland, published research output increased year-on-year from 22 papers in 2006 to 52 in 2015. The increase can be linked with several initiatives to build palliative care research capacity in Scotland. These include the establishment of two Chairs in Palliative Care at the University of Edinburgh, followed, more recently, by one at the University of Glasgow; the strengthening of palliative care research groups as a result of these positions; growth in palliative care research activity across all universities; success in obtaining funding for PhD studentships; collaboration with other specialities; greater funding opportunities in particular through charities and the Scottish Government; greater publication opportunities with the launch of the BMJ Supportive and Palliative Care in Scotland in 2010, and subsequently the launch of the BMJ Open; introduction of Research Lead posts in hospices; and the development of skills in writing, publishing and dissemination. These developments have all been supported at a higher level by the Scottish Government and the World Health Organisation advocating for evidence and research to improve integrated palliative care to all who would benefit.

### Nature of Scottish palliative care research

The 308 papers identified represent a diverse range of palliative care research activity. Several studies from the medical, nursing and allied health professions were identified, alongside research from sociological perspectives. Such diversity reflects the multidisciplinary nature of palliative care and the focus on the multidimensional needs of patients and their families [8, 45].

A large proportion of research identified was descriptive in nature (73%). A similar finding was reported in the Irish and Swedish reviews [5, 46]. Descriptive studies provide evidence on needs, experiences, perspectives and contexts to inform the design and development of interventions [47], but as evidence in a field increases evidence synthesis is required. In the present Scottish review, there was a strong focus on evidence synthesis with a relatively large number of review papers identified (14%) compared with Ireland (4%) and Sweden (4.5%). Reviews are vital in summarising the evidence base, identifying key research findings as well as gaps, and are an essential first step in intervention design. However, in Scotland, as in Ireland and Sweden, intervention studies remain under-represented and few implementation studies were identified. One possible explanation for this is that in a relatively young field like palliative care, initial studies tend to be descriptive rather than interventional; it is expected that as the field matures intervention studies will increase. Another explanation may be the cost - intervention studies generally require funding to establish and run the intervention as well as funding for its evaluation. Joint funding models which involve contributions in kind from the setting in which the intervention is embedded, as well as funding for the evaluation are needed.

In terms of research design, RCTs were evident in only 4% of published papers (12 papers). This is a greater number than reported in the previous Scottish review which identified only two RCTs over a 15 year period, and the Irish review which identified only one RCT in ten years [4]. Of these 12, four involved data collection overseas with a Scotland-based researcher on the research team, and 8 involved data collection in Scotland or the wider UK. The challenges associated with RCTs in palliative care are well documented, as are recommendations for conducting such studies [48–51]. Participant recruitment and gate-keeping is a particular challenge [52], but there is growing evidence that patients with advanced illness are open to taking part in research and may even benefit from involvement [53, 54]. More recent RCT studies in Scotland have incorporated a qualitative research component alongside traditional quantitative data collection [55]. Such studies have great potential value in providing evidence not only on intervention effectiveness but also on acceptability of the intervention for participants’ and their families.

### Research involving people with non-malignant disease

Our review reveals a substantial quantity of research focused on palliative care for people with a non-malignant diagnosis. Studies on palliative care for people with an illness other than cancer accounted for nearly one-fifth of all publications in the review period (58 publications). This is a significant number considering that 48% of all papers did not specifically state whether the participants had cancer or not. The growth in palliative care research for people with a non-malignant diagnosis is apparent in other countries, but to a lesser extent than in Scotland. In a Swedish review covering 2007 to 2012, the proportion of studies involving patients with a noncancer diagnosis was 13%, up from 9% compared with a previous review [5]. In Ireland there was also an increase in palliative care research for people with a non-cancer diagnosis, though the exact proportion was not reported [4]. This trend mirrors the growing consensus that palliative care should be integrated into care for people with any advanced progressive illness [56]. Scotland-based studies have focused on palliative care for people with heart failure [10, 25, 57], COPD [58–60] advanced liver disease [25, 61, 62] and multimorbidities [63, 64]. Carer perspectives have also been routinely gathered [10, 25, 64, 65]. Scottish research has also shown that people with non-malignant conditions are less likely to be identified for a palliative care approach [47, 66]. The Supportive and Palliative Care Indicators Tool (SPICT) has been developed to improve identification of people for palliative care, irrespective of diagnosis [67]. It has been translated in eight languages and now used internationally to improve identification, assessment and planning for a palliative approach [68]. Evaluation studies are in progress.

### Research to inform policy, service development and clinical practice

In Scotland, researchers have a strong track record in relation to engagement with policy-makers at a national level. Two national strategies for palliative and end of life care have been published in the last decade and both have drawn on Scottish palliative care research [1, 69]. Living and Dying Well, the national plan for end of life care in Scotland (2008), drew on illness trajectory work to describe how an understanding of the different pathways experienced by patients with different conditions could be helpful in planning services more appropriately to meet patients’ and carers’ needs [70, 71]. The Palliative and End of Life Care Strategic Framework for Action (2016–21) draws on a broad range of evidence generated by Scottish research groups during the period covered by this review [3]. This includes research evidence on palliative care need in the hospital population [9]; inequalities in identification for and access to palliative care [47, 66, 72]; approaches to identifying people for a palliative approach [67], improving palliative care in care homes [73, 74], understanding differing experiences and needs [75], and meeting the psychological and social needs of people affected by terminal illness [12]. The value placed on research evidence in Scotland is highlighted by the inclusion of the fifth commitment within the current national strategy to strengthen research and knowledge exchange through the establishment of a Scottish Research Forum in Palliative and End of Life Care [1]. This forum was formed in 2016 and brings together researchers, healthcare professionals, health service managers, social care professionals and policy-makers to promote knowledge exchange and evidence based practice in palliative care across Scotland.

The 308 papers identified in this review cover a range of services and settings, with implications for the delivery of palliative care in home, hospital, hospice and care home settings. Research findings are shared within the academic community via peer reviewed publication, conferences, and social media. However, in order to maximise impact, researchers need to go beyond the academic community to reach research users, in particular service managers and health and social care professionals. National conferences, such as those run by the Scottish Partnership for Palliative Care and Hospice UK, are not primarily research focused, but provide excellent opportunities for researchers to share research findings that will inform practice with a broad audience of practitioners. Providing opportunities for health and social care professionals to undertake research training through part-time Masters and PhD programmes is also vital in building a workforce that is aware of the importance of evidence and how it can inform service development and clinical practice.

### International reach

Scotland based researchers have led on, and contributed to, palliative care research initiatives internationally. One fifth of all publications identified included co-authors based in institutions outside of the UK, highlighting a high degree of European and international collaboration. Researchers at the University of Glasgow have led the development of approaches to map palliative care internationally and have conducted several benchmarking studies to inform palliative care policy, education and service development internationally [76–80]. Researchers at the University of Edinburgh led the EAPC Taskforce in primary palliative care to examine what facilitates and hinders the development of palliative care in the community across Europe, revealing that failure to identify people for a palliative approach is the main challenge [81]. These Scottish studies were disseminated to the World Organisation of Family Doctors (WONCA) and palliative care professionals who provided evidence for the adoption of the WHO resolution in 2014 to integrate palliative care in all settings [82]. Radical health service reformers have also cited Scottish research evidence on multidimensional needs as a useful approach when considering how to meet future palliative care need in the context of an aging population [83]. Several research projects to improve palliative care in developing countries have been led by researchers based at the Global Health Academy at the University of Edinburgh where researchers collaborate closely with academics and healthcare professionals in several African countries, and support clinicians to undertake PhD research that will impact the palliative care delivery in their region [84–89].

### Gaps in Scottish palliative care research

Despite a strong research output, some research gaps emerged: health economics, eHealth, care home research, children and young people and resilience. Few studies identified in this review included a health economics component. Health economics is now an essential aspect of any evaluation study, so collaborative studies involving health economics input are increasingly required [90]. Few studies examined the role of eHealth, despite the role that such technologies could potentially play in increasing access to palliative care, supporting people cared for at home, facilitating communication and coordination, and delivering training and palliative care education to health and social care professionals, especially in rural or geographically remote areas. Researchers in Scotland recognise the importance of the care home setting for palliative and end of life care, and are calling for the establishment of a care home centre of excellence in teaching and research [91]. However, there have been relatively few research studies on end-of-life care for people with dementia despite the growing number of people who will die with dementia in the coming years. The lack of research on palliative care for people with dementia may be due in part to the ethical and practical difficulties in this context. Children are also under-represented. Although 19 studies relating to children were identified, only four involved children themselves as participants – there is a need for studies involving children as participants to hear the voice of children and young people experiencing terminal illness, or those who have been bereaved. Despite growing interest in resilience [92], we did not identify any studies that specifically focused on resilience. Research on resilience in patients, carers and health care professionals in a Scottish context is warranted, especially in context of a gradual shift towards care in the community and need for more realistic and person-centred approaches to help patients and families [93].

Legislation requiring the integration of health and social care came into effect in Scotland in April 2016. This is a significant change to the way people in Scotland are cared for and, now more than ever, there is a need for health and social care research to describe and evaluate these changes, so that those involved in commissioning health and social care services have the evidence they need to design palliative care services to best meet the needs of the population of Scotland.

### Role of the third sector in supporting and funding research

The third sector makes a significant contribution to research in Scotland, both in terms of supporting and funding research activity. In the current review, hospices were the setting for data collection in 30 papers (10%) and supported studies on preferences for place of death, symptom management, and psychosocial support. Hospices have been encouraged to increase their investment in research support activities and become research-active [94]. While the challenges for hospice engagement in research are well-documented [95, 96], there is a growing appetite for research engagement and recognition of the need for the development of hospice-university partnerships to facilitate high quality palliative and end of life care research in Scotland.

The charitable sector has been a significant source of funding for palliative care research in Scotland and in the UK. According to an analysis of the National Cancer Research Institute (NCRI) databases, Marie Curie research funding accounted for more than 50% of the research spent on palliative and end of life care in 2012–13 [97]. Across the UK, Marie Curie funded 34 projects between 2010 and 2016; eight of these were led by Scottish Investigators. Other charitable funders of Scottish palliative care research include the Children’s Hospice Association of Scotland, Macmillan Cancer Support, Dunhill Medical Trust, Dimbleby Cancer Care and the British Heart Foundation. To support continued palliative care research progress in Scotland over the period of the Strategic Framework for Action and beyond, there is a need for governmental and research council funding to support post-doctoral and doctoral palliative care research; clinical-academic posts (Clinical Research Fellows and Research Nurses); knowledge exchange activities, and the Scottish Research Forum in Palliative and End of Life Care.

### Strengths and limitations of the review

While the current review is comprehensive, it was limited for pragmatic purposes. Our search strategy focused specifically on research published in peer-reviewed journals. Research described in unpublished or government reports and in PhD or Masters dissertations was not included (although much good quality postgraduate work is subsequently disseminated via publication). Fifty-four editorial/comment/discussion papers were identified but deemed ineligible as they were not research studies. However, these papers are important as they map out the latest developments in a field and advance debate and dissemination, and some were in high-impact generalist journals. The large numbers of editorials and discussion papers further reflect the impact of the Scottish research community on shaping the palliative care research agenda nationally and internationally.

An unrepresented area in this review is research conducted in Scotland where none of the researchers are based at a Scottish institution. An example is the Children in Scotland requiring Palliative Care (CHiSP) study funded by the Children’s Hospice Association of Scotland and conducted by researchers at the University of York. This investigated the number of children in Scotland in need of palliative care [46]. Systematic identification of such studies is impossible as it would require searching all palliative care papers and reading abstracts and full texts to identify the location of data collection. However, a small number of papers relevant to palliative care in Scotland may have been excluded because of this.

The present study was a systematic scoping review [7]. As is the case with scoping reviews, quality appraisal was not undertaken. Appraising the quality of 308 papers on diverse themes involving different methodologies would be a complex and time-consuming task requiring multiple appraisal tools and, given the diversity of research topic, may not be altogether meaningful. Future research might usefully focus on some of the key areas of research identified within this review - for example, conducting a systematic review of the research on people with non-malignant disease, including a quality appraisal of current evidence and recommendations on how the evidence might inform service innovation, policy and practice.

### Directions for future research

In 2015, following extensive consultation with patients, carers and healthcare professionals across the UK, the James Lind Alliance (JLA) Priority Setting Partnership (PeolcPSP) published a set of key priorities for palliative care research [98]. Research on interventions to deliver palliative care for people with non-malignant diseases was identified as a top ten priority (priority 6). Given the expertise in this area in Scotland, research on early palliative care interventions for people with non-cancer and multi-morbidity is warranted. In particular research on interventions to support frail/older people living with dementia, either in their own homes or in care homes are required.

Another top priority identified by the JLA is research on the information and training needs of carers and families. Scotland based researchers have a strong track record in terms of involving carers in research [10, 16, 18, 64, 65, 73, 99, 100]. Further research to enable family members to care for a terminally ill person at home, and to support their own health and wellbeing, is recommended. Such research could helpfully inform the design of community palliative care services that enable patients to remain at home for as long as they wish to.

In the current review, very few papers on out-of-hours care were identified, despite this being identified as the number one priority for research according to the JLA [98]. Given the current dearth of evidence on out-of-hours and continuity of care as well as the national focus on improving out of hours care [101], this will remain an important direction for research in the future. The crucial link between eHealth integration improvements and the quality of out-of-hours palliative care provision is also an important potential area for research.

Physical symptom assessment and management has been a key area of research activity in Scotland and continues to be a top research priority [98]. In Scotland, research on physical symptoms has focused on pain, cachexia and delirium. These symptoms are amongst the most frequently reported by patients with a terminal illness, and evidence on the best approaches to manage these symptoms continues to be a requirement. There is also a particular need for evidence relating to managing symptoms in people with dementia, Parkinson’s and other illness that affect cognition. In addition to physical symptoms, research on psychotherapeutic interventions to recognise and treat anxiety, low mood and depression in terminally ill people and their carers is needed. Other priority areas include bereavement support, support for children and young people experiencing terminal illness either themselves, or within their families and research on public health approaches to support more openness and opportunities to discuss death, dying and bereavement in the community.

It is recommended that a follow-up review is conducted in 2021, covering the five-year period of the strategic framework for action. The present review provides a baseline against which future research activity can be compared both in terms of volume of output and core research areas. Similar comparisons over time have been conducted in Sweden and Ireland, and provide useful insights into how palliative care research is developing over time in a national context, and what areas need to be prioritised for the future. Finally, we urge researchers to consider conducting similar reviews of palliative care research in their own countries or regions. This would generate research databases to inform evidence based decision-making regionally or nationally, and could help map the palliative care evidence base internationally.

## Conclusion

There is a strong, active and highly productive palliative care research community in Scotland, with a considerable increase in research output over the period 2006 to 2015, and a high volume of publications compared with similar reviews in Ireland and previously in this country. Research into the patient and carer experience of living and dying of many illnesses is internationally leading, and intervention studies are now required in all settings. Future priority areas include out-of-hours palliative care provision and access, interventions for people with non-malignant illness, dementia and multi-morbidity; physical and psychological symptom assessment and management; interventions to support carers; and bereavement support for children. Knowledge exchange activities are required to promote research into practice. Continued work on validated indicators to assess improvement in palliative care delivery locally and nationally are required. A follow-up review to track research progress and dissemination is recommended.

## Appendix 1: Medline Search Strategy

1. (edinburgh or glasgow or dundee or strathclyde or st andrews or west scotland or heriot-watt or napier or robert gordon or abertay or queen margaret or aberdeen or stirling or highlands).in.

2. (dying or bereavement or bereaved or grief or "place of death").mp. [mp=title, abstract, original title, name of substance word, subject heading word, keyword heading word, protocol supplementary concept word, rare disease supplementary concept word, unique identifier]

3. (palliative or palliate or palliation or hospice or "end of life" or "last year of life").mp. [mp=title, abstract, original title, name of substance word, subject heading word, keyword heading word, protocol supplementary concept word, rare disease supplementary concept word, unique identifier]

4. (care home* or nursing home*).mp. [mp=title, abstract, original title, name of substance word, subject heading word, keyword heading word, protocol supplementary concept word, rare disease supplementary concept word, unique identifier]

5. ("supportive care" or "supportive oncology").mp. [mp=title, abstract, original title, name of substance word, subject heading word, keyword heading word, protocol supplementary concept word, rare disease supplementary concept word, unique identifier]

6. ("terminal care" or "terminal illness").mp. [mp=title, abstract, original title, name of substance word, subject heading word, keyword heading word, protocol supplementary concept word, rare disease supplementary concept word, unique identifier]

7. ((Advanced or late-stage or end-stage) and (disease or illness or cancer or carcinoma or neoplasm) and (symptom control or pain or cachexia or breathlessness or nausea or distress)).mp. [mp=title, abstract, original title, name of substance word, subject heading word, keyword heading word, protocol supplementary concept word, rare disease supplementary concept word, unique identifier]

8. (End-stage and (heart or liver or cardiac or kidney or renal)).mp. [mp=title, abstract, original title, name of substance word, subject heading word, keyword heading word, protocol supplementary concept word, rare disease supplementary concept word, unique identifier]

9. (dementia and (advanced or late-stage)).mp. [mp=title, abstract, original title, name of substance word, subject heading word, keyword heading word, protocol supplementary concept word, rare disease supplementary concept word, unique identifier]

10. Palliative Care/ or "Hospice and Palliative Care Nursing"/ or Palliative Medicine/

11. 2 or 3 or 4 or 5 or 6 or 7 or 8 or 9 or 10

12. (scotland or scottish).mp. [mp=title, abstract, original title, name of substance word, subject heading word, keyword heading word, protocol supplementary concept word, rare disease supplementary concept word, unique identifier]

13. 1 or 12

14. 11 and 13

15. 14

16. limit 15 to yr="2006 -Current"

## Additional file

Additional File 1: Summary of findings from included publications (*n* = 308). This file presents the data extracted from each of the 308 publications identified in this review. (XLSX 163 kb)

## Abbreviations

AIIHPC: All Ireland Institute of Hospice and Palliative Care
COPD: Chronic obstructive pulmonary disease
CSO: Chief Scientist Office
EAPC: European Association for Palliative Care
JLA: James Lind Alliance
NHS: National Health Service
NIHR: National Institute for Health Research
RCT: Randomised controlled trial
